# APPlications of amyloid-β precursor protein metabolites in macrocephaly and autism spectrum disorder

**DOI:** 10.3389/fnmol.2023.1201744

**Published:** 2023-09-20

**Authors:** Deborah K. Sokol, Debomoy K. Lahiri

**Affiliations:** ^1^Department of Neurology, Section of Pediatrics, Indiana University School of Medicine, Indianapolis, IN, United States; ^2^Department of Psychiatry, Indiana University School of Medicine, Indianapolis, IN, United States; ^3^Department of Medical and Molecular Genetics, Indiana University School of Medicine, Indianapolis, IN, United States; ^4^Indiana Alzheimer Disease Research Center, Indiana University School of Medicine, Indianapolis, IN, United States

**Keywords:** ASD, macrocephaly, amyloid precursor protein, adamalysins, brain development, overgrowth

## Abstract

Metabolites of the Amyloid-β precursor protein (APP) proteolysis may underlie brain overgrowth in Autism Spectrum Disorder (ASD). We have found elevated APP metabolites (total APP, secreted (s) APPα, and α-secretase adamalysins in the plasma and brain tissue of children with ASD). In this review, we highlight several lines of evidence supporting APP metabolites’ potential contribution to macrocephaly in ASD. First, APP appears early in corticogenesis, placing APP in a prime position to accelerate growth in neurons and glia. APP metabolites are upregulated in neuroinflammation, another potential contributor to excessive brain growth in ASD. APP metabolites appear to directly affect translational signaling pathways, which have been linked to single gene forms of syndromic ASD (Fragile X Syndrome, PTEN, Tuberous Sclerosis Complex). Finally, APP metabolites, and microRNA, which regulates APP expression, may contribute to ASD brain overgrowth, particularly increased white matter, through ERK receptor activation on the PI3K/Akt/mTOR/Rho GTPase pathway, favoring myelination.

## Introduction

We describe a chronicle primarily of four ‘A’s (APP, AD, ADAMs, and ASD) leveraging biochemistry and neurology and linking brain developmental events across the human life span. Amyloid-β Precursor Protein (APP) is a transmembrane glycoprotein expressed at high levels in the brain (O'Brien and Wong, 2011). Its proteolytic product, amyloid-β peptide (Aβ) has been widely studied as a causal component of Alzheimer’s Disease (AD). However, APP appears to contribute to other physiological activities, including neurogenesis, neurite outgrowth, synaptic plasticity, and neuroprotection (Luu et al., 2021). Spanning the membrane, APP undergoes proteolytic cleavage in its ectodomain by either α- or β-secretase, or in the transmembrane by γ-secretase complex. Once cleaved, APP metabolites are released in secreted (s) forms. Initiation of the catabolic amyloidogenic pathway generates Aβ peptides that comprise the cerebral plaques associated with brain atrophy found in AD. Following the amyloidogenic pathway, there is sequential cleavage of APP by β-secretase (BACE1), which generates the larger APP segment (APPβ) and a carboxy-terminal fragment, subsequently split by γ-secretase. This generates neurotoxic Aβ peptides 40 and 42 residues (Aβ40, Aβ42), and the APP intracellular domain (AIDC). However, the anabolic, nonamyloidogenic α-secretase pathway is the constitutive, physiologic pathway. Along this pathway, APP is cleaved via the A Disintegrin and metalloproteinase enzyme (adamalysins, ADAM 10/ 9/17), secreting sAPPα (Hardy, 2009). It has been demonstrated that sAPPα has neurotrophic properties on neurons (Mattson, 1994; Ray et al., 2011) and glia (Baratchi et al., 2012). The remaining intramembrane protein is cut by γ-secretase producing the smaller P3 peptide and AICD.

APP and its metabolites are dysregulated in ASD (Lahiri et al., 2013). We and others have reported high levels of total sAPP and sAPP a (Sokol et al., 2006; Bailey et al., 2008; Sokol et al, 2009; Ray et al., 2011; Frackowiak et al., 2013; Ray et al., 2016) and low levels of Aβ peptides (Aβ40, Aβ42) in plasma and brain tissue from children with ASD (Ray et al., 2016). Recently, higher levels of plasma total sAPP and sAPP a were found for children with regressive ASD compared to those with ASD but no regression (Li X. et al., 2022). ADAM17 is one of the α-secretases that cleaves full-length APP to sAPPα (Buxbaum et al., 1998; Bandyopadhyay et al., 2007). Considered proinflammatory, ADAM17 is also called tumor necrosis factor-α converting enzyme (TACE). It releases the cytokine, tumor necrosis factor-α (TNF-α; Kim et al., 2008). Interestingly, in murine models, ADAM17 and ADAM10 were shown to be required to develop oligodendrocytes (Sakry et al., 2014; Palazuelos et al., 2015), the biggest producers of myelin within the CNS. Notably, we found increased levels of ADAM17 in temporal lobe brain tissue from children with ASD (Ray et al., 2016).

Understanding the relationship of APP metabolites to other related disorders has also been our major focus. We found a fascinating profile of APP metabolites in plasma from children with Fragile X Syndrome (FXS) and adult FXS brain tissue: increased levels of total APP, sAPPα, and Aβ peptide (Ray et al., 2016). Although FXS is often associated with ASD symptoms, this difference illustrates a complex relationship between APP metabolites, and disorders with ASD behavioral symptoms. Altogether, these results are consistent with the “Anabolic Hypothesis” of APP in ASD (Sokol et al., 2011; Lahiri et al., 2013), and the potential contribution of APP metabolites to brain enlargement in this disorder.

In this review, we discuss normal brain growth, and brain growth contributing to macrocephaly in ASD and the potential application of APP metabolites to general brain formation. We examine how neuroinflammation and translational signaling pathways could result in brain, predominantly white matter enlargement in ASD. We propose an integrated pathway that links sAPPα to the ERK1/2 → MAPK → PI3K/AKT–mTOR → Rho GTPases pathway, favoring increased myelination, seen in ASD.

## Normal brain growth and brain cell differentiation involve APP

Brain development begins with neural progenitor cell differentiation which takes place in the third week of gestation, then continues through late adolescence and in some locations, throughout the lifespan. Brain size depends on the combined cortical gray and subcortical white matter volumes (Ohta et al., 2016). The cortical gray matter gets its color from and is composed of neuron cell bodies and dendrites, while the subcortical white matter is colored by white myelinated axons projecting downward from the outer layer. Gray cerebral cortical volume is determined by cortical surface and thickness, two physical features that have been found to be genetically independent (Panizzon et al., 2009). Rakic (1988) first theorized that cortical surface area and thickness associate with radial cortical columns. The number of columns determines cortical surface area, while the number of cells within the columns determines cortical thickness (Rakic, 1988). The columns themselves originate from founder progenitor cells that symmetrically divide in the subventricular zone (SVZ) before neurogenesis (Ohta et al., 2016). This exponentially increases the number of potential columns determining the amount of cortical surface (Ohta et al., 2016). After the final cell division in the SVZ, neurons migrate along a common radial glia fiber to the cortex (Ohta et al., 2016). Over half the human brain consists of white matter, which is of larger proportion in humans compared to other animals (Fields, 2008). Essential for cognitive function, the myelinated white matter enables rapid action potential propagation (Courchesne et al., 2003; Herbert, 2005; Dawson et al., 2007; Fields, 2008). White matter consists of myelinated axons and glia. There are three types of glia: (1) astrocyte glia establish the blood brain barrier and support synaptic transmission (2) microglia provide host defense and tissue repair and are the primary source of cytokine release, and (3) oligodendrocytes insulate the axon by forming myelin sheaths (Jones, 2014).

Glia exist in both gray and white matter, but oligodendrocytes are more abundant in white matter (Dimou et al., 2008). Oligodendrocyte precursor cells (OPCs), are specialized glia that give rise to oligodendrocytes. Further, OPCs “talk back to neurons” by producing neuron/glia antigen 2 (NG2; Jones, 2014). NG2^+^ cells are often found directly contacting neurons (Karram et al., 2005). This cellular communication requires complex but coordinated biochemical events. NG2 glia direct OPC polarity and migration via Rho GTPase family members RhoA and Rac1 signaling (Biname et al., 2013). As described below, the mTOR pathway activates cofilin through Rac1, which controls myelination; Wnt signaling guides OPC embryonic development as OPCs traverse endothelial cells (Tsai et al., 2016).

Murine models support the role of APP, and in particular, sAPPα, in neurogenesis and neuronal differentiation (Zhou et al., 2011; Luu et al., 2021). Transgenic mice overproducing human APP have 10–15% greater cortical neurons than wild type mice (Bondolfi et al., 2002). APP levels peak at postnatal day 24, coinciding with brain maturation (Loffler and Huber, 1992). APP downregulation was shown to inhibit neural progenitor cells (NPCs) from the SVZ to the cortical plate in the developing cortex, an effect rescued by inserting full length APP into the NPCs (Young-Pearse et al., 2007). Overexpression of APP (as expected in ASD) accelerated NPC migration into and beyond the cortical plate (Young-Pearse et al., 2007). Acting anabolically, secreted APP (including sAPPα) was shown to bind epidermal growth factor (EGF) progenitor cells, which induced neurospheres within adult rodent SVZ (Caillé et al., 2004). Decreasing sAPP by inhibiting α-secretase reduced cell proliferation in the adult rodent SVZ. This was rescued via sAPP, demonstrating sAPP’s direct impact on stem cell proliferation (Caillé et al., 2004). These studies demonstrate how APP may contribute to embryonic and adult neurogenesis.

Detailed mechanistic studies of the effect of APP metabolites on neurogenesis have been made possible with the study of induced pluripotent stem cells (iPSCs). APP expression and proteolytic processing was studied over 100 days using human iPSCs (Bergström et al., 2016). Notably, sAPPα was secreted from neural progenitors early in differentiation. β-cleaved soluble APP (sAPPβ) came later after several layers of neurons were produced. Short Aβ peptides peaked during the progenitor stage while longer Aβ 40/42 peptides (associated with AD) processing occurred when post-mitotic neurons appeared. This suggests that APP metabolites expression follows a developmental pattern and that not only anabolic sAPPα, but also Aβ 40/42 peptides, seen in AD, contribute to the brain growth (Bergström et al., 2016). It has been long suspected that Aβ 40/42 peptides must serve another purpose besides giving rise to AD (Lahiri and Maloney, 2010).

## ASD and macrocephaly

Eighty years ago, Leo Kanner noted that the children he described with ASD often had large heads (Kanner, 1943). Both enlarged *heads*, or macrocephaly, as determined by occipital-frontal circumference (OFC), and enlarged *brains*, or megalencephaly, as determined by MRI or *post mortem* analysis, are reported in ASD. It is generally agreed 15%–20% of children with ASD show macrocephaly where OFC is 2 standard deviations above the mean based on age and gender (Fombonne, 1999; Nordahl et al., 2011). Early studies showing larger OFC in children with ASD have been criticized due to insufficient sampling, the use of misleading population norms and differing growth curves (Daymont et al., 2010; Raznahan et al., 2013). However, other studies have shown that short of falling on the extreme end of the curve, more children with ASD have larger heads than children with typical development (Sacco et al., 2015). Boys with ASD have larger heads than girls with ASD (Nordahl et al., 2011; Lee et al., 2021), and boys with enlarged brains usually have more severe ASD (Amaral et al., 2017). Early brain enlargement is seen by age 4, coincidental with the display of ASD symptoms (Courchesne et al., 2001; Aylward et al., 2002; Sparks et al., 2002; Stanfield et al., 2008; Nordahl et al., 2011) and continues up to age 12, as reported in a recent longitudinal study (Lee et al., 2021). Several of these studies have reported increased total cerebral volume (Nordahl et al., 2011; Amaral et al., 2017; Pagnozzi et al., 2018), cortical thickness (Raznahan et al., 2013), cortical surface area (Hazlett et al., 2011; Ohta et al., 2016), and regional gray (Casanova et al., 2006; Hyde et al., 2010; Amaral et al., 2017), and white matter associated with ASD (Courchesne and Pierce, 2005; Herbert, 2005). The functional correlates of increased brain growth, particularly for boys with ASD, include social dysfunction (Lainhart et al., 1997), regression (Nordahl et al., 2011), more severe disabilities/poorer prognosis (Amaral et al., 2017), and language deficits (Naigles et al., 2017). A particular cohort of boys with “disproportionate megalencephaly” has been identified who have larger head to height ratios and more severe cognitive impairment (Nordahl et al., 2011; Lee et al., 2021).

ASD has been described as a disorder of brain connectivity with intact cortical gray matter regions, i.e., visual cortex, but disordered white matter connections between cortical regions (Minshew and Williams, 2007). In ASD, increased white matter exists in specific regions (Boddaert et al., 2004; Waiter et al., 2005; Ke et al., 2009; Lainhart, 2015), in outer zones (Herbert et al., 2004), and the whole brain (Courchesne et al., 2001). Hypermetabolism, as measured by Fluorodeoxyglucose positron emission tomography (PET), was determined for white matter tracts from the corpus callosum, internal capsule, and frontal and temporal lobes of young adults with ASD, similar to PET results seen in subjects with schizophrenia (Mitelman et al., 2017). These findings were explained as a result of a disruption in myelin (Mitelman et al., 2017).

Diffusion weighted MRI imaging (DW-MRI) has enabled the assessment of white matter microstructures using fractional anisotropy (FA) as a measure of myelination. A recent longitudinal study showed that young children with ASD were slower to develop fractional anisotropy (FA) than typically developing controls (Andrews et al., 2021). Further, children with severe ASD had a slower developmental trajectory of FA within the sagittal stratum, showing a functional correlate, i.e., ASD severity, of the reduced FA trajectory. A few studies have suggested that increased FA may precede a later decrease in FA (Wolff et al., 2012; Solso et al., 2016). Accelerated cortical growth in ASD may result in increased axonal projections and densities with disrupted neural networks, resulting in an initial increase, followed by the eventual disruption/loss of myelin (Andrews et al., 2021). It may be more accurate to assume that excess in white (or gray) matter seen in ASD, although looking like normal tissue, may not function normally.

## Macrocephaly, neuroinflammation, and APP

Neuroinflammation (including astrogliosis and microgliosis), rather than an increase in neurons and axons, could drive white matter enlargement in ASD (Herbert, 2005; Vargas et al., 2005; Fields, 2008; Aoki et al., 2017; Lee A. S. et al., 2017). Neuroinflammation can be seen microscopically, but signs of inflammation are not seen on brain MRI in ASD, which parallels findings in AD (Herbert, 2005). Immunochemistry has uncovered an increase of cytokines within the cerebrospinal fluid (CSF) and microglia-astroglia activation within the amygdala, dorsolateral prefrontal cortex, and cerebellum in adults and children with ASD (Vargas et al., 2005; Casanova et al., 2006; Morgan et al., 2010, 2014).

Activated microglia and astroglia, with resultant cell swelling and tissue volume increase, or concurrent increase in vascularity, could account for subtle overall and white matter volume increase seen in ASD (Herbert, 2005). Neuroinflammation may activate chemokine or cytokine immune function modulators, as identified in a longitudinal study of serum and CSF from young children with ASD (Pardo et al., 2017). Soluble CD40 ligand and EGF, both immune function modulators, were increased in ASD serum (Pardo et al., 2017). This result was thought to be due to genetic dysregulation as the researchers found no active systemic inflammatory response such as elevated erythrocyte sedimentation rate (Pardo et al., 2017). The CSF immune mediators CCL2, CX3CL, CXCL1, FLT3L, and IL-15 were elevated, explained as a genetic “homeostatic” support of microglia instead of an inflammatory reaction to infection or disease.

APP is upregulated during inflammation, and proinflammatory astrocytes and cytokines activate it (Lahiri et al., 2003; Herbst-Robinson et al., 2015; Hefter and Draguhn, 2017). Aβ peptides and sAPPα both are stimulated via glia, and APP contributes to the acute phase protein immune response to stress (Yang et al., 2015). sAPPα appears gliogenic, triggering the release of glutamate, IL-1, and inflammatory markers in microglia (Barger and Harmon, 1997; Li et al., 2000; Barger and Basile, 2001). When treated with recombinant sAPPα, neural stem cells (Kwak et al., 2006) and differentiated NPCs produce abundant astroglia (Baratchi et al., 2012). In a study of the brain confocal microscopy, both children with idiopathic ASD and those with dup15q11.2-q13 showed increased intraneuronal accumulation of the P3 peptide aggregates in astrocytes and microglia. P3 is a product of the nonamyloidogenic pathway that also produces sAPPα. Higher levels of P3 equivalent were identified by specific antibodies within gray and white matter regions that differed by group: highest for dup15q11.2-q13, next for idiopathic ASD, and last for typically developing controls (Wegiel et al., 2012). Epilepsy and sudden death was also elevated in the dup15q11.2-q13 group, which raised the possibility that besides activation of astroglia, products of the nonamyloidogenic pathway may contribute to epilepsy, a common co-morbidity in ASD (Wegiel et al., 2012). We wish to avoid giving the impression that APP expression is increased in all conditions. Several factors, such as treatment of neuronal culture with pineal hormone melatonin decreased APP secretion (Song and Lahiri, 1997), and specific cholinesterase inhibitors, reduced levels of APP and Aβ peptides (Lahiri et al., 1994). Recent studies have shown that non-coding RNAs, specifically microRNAs (miRNA), alter APP expression; e.g., miR-101 and miR-20b independently regulate APP metabolites (Long and Lahiri, 2011; Wang et al., 2022). Further studies are warranted to see levels of miR-101 and miR-20b in ASD subjects. We will discuss other miRNAs later. In addition, mis-regulation of APP mRNA transport, translation and processing in relation to ASD is another area of further research (Lahiri et al., 2021).

Early murine studies of APP reveal that increased APP accumulation and immunoreactivity is the expected outcome in white matter after focal cerebral ischemia (Yam et al., 1997). Further, APP was found to accumulate in the brains of patients who died after head injury (Sherriff et al., 1994). APP accumulation was attributed to trauma to the axon, preventing axoplasmic flow as APP is present within axonal microtubules and is transported by fast anterograde axonal transport (Yam et al., 1997). APP accumulation was more prevalent in mild cases of white matter ischemia compared to moderate and severe cases of white matter injury, suggesting that APP immunoreactivity occurs in the early stage of white matter lesions. Further, “recent studies have shown abnormal white matter in every AD brain” (Sharp et al., 2023). Although the characteristic AD plaques and tangles are found in gray matter, increased Aβ 40/42 peptides damage myelin, causing cholesterol release and cholesterol dysmetabolism contributing to Aβ dysmetabolism, and a vicious injury cycle (Sharp et al., 2023).

It is also possible that elevation in APP metabolites in the autistic brain is associated with injury, such as head banging, which is often seen in individuals with severe ASD. An increase in APP, particularly sAPPα, found in traumatically injured brains may be a neuroprotective response. Animal studies have shown naturally increased levels of brain sAPPα after head injury and improved axonal injury and motor outcome after exogenous administration of sAPPα (Marmarou et al., 1994; Thornton et al., 2006).

## Adamalysins’ role in inflammation, cellular growth, and myelination

ADAM secretases (adamalysins) ADAM 9, 10, and 17 constitutively cleave APP to produce sAPPα, while subsequent γ-secretase cleavage produces AICD and P3 peptide following the anabolic, non-amyloidogenic pathway. However, ADAM17 cleaves proteins other than APP, acting as an extracellular convertase that promotes cellular growth and cytokine signaling during inflammation. Lipopolysaccharide (LPS), existing within bacteria cell wall, and other toxins stimulate the release of interleukins [e.g., IL 1-β and receptor (R)]. ADAM17 mediates shedding of several interleukins (de Queiroz et al., 2020), along with regulating proinflammatory tumor necrosis factor (TNF) and tumor necrosis factor receptor (TNFR; McMahan et al., 2013). Participating in cellular growth, ADAMs have both cell adhesive and proteolytic properties that effect cell surface molecules participating in cell adhesion and with epithelial growth factors (EGF): Transforming growth factor alpha (TGF-α), and its EGF receptor (R; Blobel, 2005; Gooz, 2010). The transforming growth factor-beta (TGF-β) receptor type 1 (TGFBR1) transmits signals from the cell surface into the cell via signal transduction that stimulates cell growth. ADAM17 appears to regulate TGFβ signaling (Kawasaki et al., 2014). ADAMs 10 and 17 cleave Notch proteins, which trigger Notch signaling, important in NPC development and differentiation (Groot and Vooijs, 2012; Matthews et al., 2017). The anabolic function of ADAMs 10 and 17 has been studied in cancer (Huang et al., 2014), spinal cord injury (Wei et al., 2015), and rheumatoid arthritis (Isozaki et al., 2013). Besides converting EGF (Lee et al., 2003), ADAM17 also converts the ASD CSF-associated cytokine mediators cited above: FLT3 (Horiuchi et al., 2009), CX3CL1 (Wong et al., 2014), CXCL8 (Ovrevik et al., 2011), and IL-15 (Khawam et al., 2009).

Adamalysins perform many physiological functions and play a role in CNS myelination. Unlike its role in inhibiting peripheral nervous system Schwann cell myelination (la Marca et al., 2011), ADAM17 is “essential” for developing oligodendrocytes and myelination in the CNS (Palazuelos et al., 2015). In transgenic mice, ADAM17 cleaves EGFR ligands, enabling activation and support of oligodendrocyte cell survival during subcortical white matter development (Palazuelos et al., 2015). EGFR overexpression in ADAM17-deficient OCPs restored cell survival, proliferation and finally, myelination (Palazuelos et al., 2015). In murine models, ADAM10 deficiency decreased glial differentiation leading to a fewer oligodendrocytes and glial cells (Saftig and Lichtenthaler, 2015).

## MicroRNA and α-secretase in ASD

Members of the small non-coding microRNA (miRNA) group regulate multiple biological and pathological processes so that disruption of miRNAs could contribute to several diseases, including ASD. MiRNAs bind a specific target mRNA sequence at its untranslated region (UTR), largely disrupting target mRNA stability and ultimately regulating protein production in translation (Luu et al., 2021). miRNAs play important roles during development and homeostasis and contribute to different cellular processes, such as cell proliferation, differentiation and apoptosis (Vidigal and Ventura, 2015; Schepici et al., 2019). MiRNA dysregulation occurs in ASD (Gandal et al., 2016; Nadim et al., 2017), and some of these same miRs have been found to regulate α-secretases associated with APP (Table 1) and/or AD. For example, miR 107, miR 144-3p, miR 448, and miR 103a-3P are dysregulated in ASD brain (Mor et al., 2015; Wu et al., 2016; Huang et al., 2021), and lymphocytes (Sarachana et al., 2010), and (normally) reduce α-secretase ADAM 10 (Augustin et al., 2012; Jiao et al., 2017; Wang et al., 2020). Resultant ADAM 10 increase, therefore, favors increased sAPPα. Likewise, miR 145-5p, is downregulated in ASD blood (Vaccaro et al., 2018), miR 338-3p is downregulated in ASD brain (Li J. et al., 2022), and (normally) both decrease α-secretase ADAM17 (Doberstein et al., 2013; Chen et al., 2015). Therefore, increase in ADAM17 favors increased sAPPα. Finally, miR 146a, upregulation occurs in ASD brain (Mor et al., 2015; Nguyen et al., 2018) and olfactory mucosa (Nguyen et al., 2016), and is the “most common miRNA deregulation in neurodevelopmental disorders such as ASD”(Li J. et al., 2022). miR146a also is dysregulated in AD (Wang et al., 2019) and in brain of AD mouse models, as reported by Lahiri et al. (2018).

**Table 1:** Validated miRNA species that target ADAM9, 10, or 17 (Mor et al., 2015; Chou et al., 2018).

miRNA	Target(s)	miRNA	Target(s)	miRNA	Target(s)
miR-26a-5p	ADAM17	miR-126-5p	ADAM9	miR-448	ADAM10
miR-33a-5p	ADAM9	miR-144	ADAM10	miR-449a	ADAM10
miR-103a-3p	ADAM10	miR-145-5p	ADAM17	miR-451a	ADAM10
miR-122-5p	ADAM10, ADAM17	miR-152-3p	ADAM17	miR-655-3p	ADAM10
miR-126-3p	ADAM9	miR-338-3p	ADAM17		

A few of the miRNAs cited above (miR338 and miR146), recently have been shown to promote oligodendrocyte differentiation (Elbaz and Popko, 2019), which may favor enlargement in white matter.

## Metabolic translational pathways contribute to macrocephaly

It has been hypothesized that ASD results in disruption of the neuronal activity that regulates mRNA translation and synaptic plasticity, as represented by many single gene ASD syndromes associated with disruption of mRNA translation (e.g., PTEN, FXS, Tuberous Sclerosis Complex-TSC, and Neurofibromatosis-NF; Kelleher and Bear, 2008; Ebert and Greenberg, 2013). Of note, macrocephaly is associated with each of these conditions.

It is worthwhile to review the translational signaling pathways that contribute to brain growth in ASD and atrophy in AD which are disrupted in these neurological conditions (Kelleher and Bear, 2008; Cai et al., 2012; Zhang et al., 2018).

Neurotrophin growth factors, including nerve growth factor (NGF) and brain derived neurotrophic factor (BDNF) bind to activate the tropomyosin-related kinase (Trk) family of receptor tyrosine kinases (TrkA, TrkB, and TrkC), which subsequently activate mammalian target rapamycin (mTOR), PI3K and extracellular signal-regulated kinase 1/2 (ERK1/2) pathways (Subramanian et al., 2015). Consistent with their purported role in controlling neurogenesis and neuronal survival, dysfunction in neurotrophin signaling has been implicated in ASD (Sadakata and Furuichi, 2009; Ray et al., 2011) and AD (Allen et al., 2011). Consistent with their role in promoting growth, tyrosine kinase receptor ligands, including NGF, increase anabolic sAPPα (Mills and Reiner, 1999).

The mammalian target of rapamycin (mTOR) is a central regulator of interlinked signaling pathways controlling cell growth, protein synthesis and cytoskeleton organization, including myelination (Laplante and Sabatini, 2009; Figlia et al., 2018). mTOR is the core component of protein complexes mTOR complex 1 and mTOR complex 2 (MTORC1, MTORC2), which control many cellular processes. Akt, a protein kinase, is an important gate-keeper to the onset of the mTOR pathway, as Akt activation targets Tuberous Sclerosis Complexes 1 and 2 (TSC1 and TSC2) and regulates mTORC1 activity (Dan et al., 2014). Activation of mTORC1 triggers the eIF4E and S6K growth factor cascade. Akt also activates the PI-3-kinase pathway that stimulates mTORC2, which controls and maintains the actin cytoskeleton via Rho GTPace family members RhoA and Rac1 signaling (Biname et al., 2013). As described below, the mTOR pathway regulates myelination by disassembling actin via activation of cofilin through Rac1 (Lipton and Sahin, 2014).

In the brain, mTOR regulates synaptogenesis and neurogenesis; the malfunction of upstream or downstream mTOR cells has been associated with AD (Cai et al., 2015) and ASD (Ganesan et al., 2019). mTORC1 activity was reduced in human AD and transgenic AD rodent brain samples, demonstrating decreased cell soma (Lee K. H. et al., 2017). In contrast to AD, mutations in TSC1 and TSC2 and hyperactivity of the Akt/PI-3kinase pathway can lead to megalencephaly and dysmorphisms/overconnectivity of developing neurons, glia, and progenitor cells seen in syndromic (TSC) and idiopathic autism (Takei and Nawa, 2014).

As members of the Mitogen-activated protein kinase (MAPK) signaling cascade, ERK 1/2 molecules transduce signals via sequential phosphorylation from cell surface receptors to the nucleus (Subramanian et al., 2015). ERK 1/2 contributes to neurogenesis by proliferating neural progenitor cells (Samuels et al., 2009). ERK 1/2 is linked to ASD as several gene mutations which cause monogenic disorders associated with ASD, including TSC, FXS, and Neurofibromatosis 1, activate ERK 1/2 (Kelleher and Bear, 2008). Further, copy number variation (CNV) and genome-wide association studies (GWAS) have identified an enrichment of MAPK/ERK signaling cascade in individuals with ASD (Pinto et al., 2010). ERK and p38MAPK-activated protein kinases directly activate ADAM17 in response to stress or inflammation signals (Xu and Derynck, 2010; Arkun and Yasemi, 2018). Recent evidence suggests that overactivation of ERK1/2 contributes to hyperphosphorylation of tau protein in AD and that suppression of ERK 1/2 phosphorylation may prove a valuable target in AD (Muraleva et al., 2021).

PI3K-Akt–mTOR pathway is a major intracellular network that increases cell proliferation as activated Akt directly phosphorylates mTOR to promote the binding of cyclin-dependent kinase that regulates cell division. Overactivation of this pathway is implicated in cancer and human tumors (Barra et al., 2019). Phosphatase and tensin homolog deleted on chromosome 10 (PTEN) is a tumor suppressor phosphatase encoded by the PTEN gene. PTEN is a negative regulator of the PI3K-Akt–mTOR pathway. PTEN genetic disorder is associated with macrocephaly, autism, and a propensity to develop tumors. Furthermore, whole exome sequencing of patients with macrocephaly and autism has uncovered mutations other than PTEN that localize to the PI3K-Akt–mTOR pathway (Yeung et al., 2017). This suggests that disruption in the PI3K-Akt–mTOR pathway contributes to macrocephaly in autism. An increase in the expression of PTEN was found for APP transgenic AD mice. This effect was reduced via a PTEN inhibitor, which also induced Akt phosphorylation, reducing the apoptotic effect in the brains of these transgenic AD mice (Cui et al., 2017).

The Wingless and integration site (Wnt) pathway is another specific signaling pathway that shows a convergence of multiple risk genes associated with autism (Kwan et al., 2016). For example, CHD8, CTNNB1(β-catenin), and PTEN gene mutations have been associated with ASD, and they also regulate Wnt proteins (Kwan et al., 2016). The Wnt pathway consists of a group of glycoproteins that transmit signals from the cell surface receptor to the nucleus, where transcription occurs (Soomro et al., 2018). Among its many functions, the signaling molecules in the Wnt family mediate activity-dependent synaptic growth and function (Budnik and Salinas, 2011). There are two main types of Wnt signaling: the canonical pathway that involves β-catenin protein and contributes to cell proliferation, cell polarity, and cell migration (and in cancer, metastasis), and the “non-canonical” β-catenin-independent pathway that participates in the actin cytoskeleton, cell adhesion, and migration (Soomro et al., 2018).

Following the canonical pathway, Wnt glycoproteins bind to the cell membrane receptor Frizzled and to the co-receptors, low-density lipoprotein receptor-related proteins 5 and 6 (LRP5/6; He et al., 2004). Wnt binding dislodges β-catenin from its cytoplasmic degradation complex, which comprises scaffolding protein Axin, glycogen synthase kinase 3 (GSK3), casein kinase 1 (CK1), and adenomatous polyposis coli (APC; Zhang et al., 2014). Subsequently, stabilized β-catenin enters the nucleus where it functions as a transcriptional activator (Zhang et al., 2014). Activation of the canonical Wnt pathway has downstream effects on other signaling cascades, including PI3K/Akt/mTOR (Hodges et al., 2018). Likewise, mTOR affects the Wnt pathway. Tumor suppressor complex (TSC1/TSC2) proteins associate with Axin and β-catenin in the canonical Wnt pathway, and an absence of either TSC1 or TSC2 increases Wnt signaling (Orlova and Crino, 2010). It appears that hypoactivity and hyperactivity of Wnt signaling can lead to cognitive dysfunction (Hodges et al., 2018).

β-catenin, the central protein of the Wnt canonical signaling pathway, impacts cortical (gray) surface area development. β-catenin transgenic mice produce a greater than usual number of NPC within the SVZ, which increases brain surface area and the number of radial columns (Chenn and Walsh, 2003). Further, the Wnt canonical pathway is critical in developing OPCs. Wnt is transiently activated in OPCs at initial differentiation and then down-regulated when oligodendrocytes achieve maturity (Soomro et al., 2018). Wnt may provide the vasculature and polarity used by OPCs to migrate to their subcortical destinations (Tsai et al., 2016).

APP has been implicated as a Wnt receptor, with a recent study showing APP binds both canonical and noncanonical Wnt ligands at a cysteine-rich domain (Liu et al., 2021). Canonical Wnt signaling dysfunction appears to hasten the onset of AD, while Wnt canonical activation may be protective against Aβ toxicity (Tapia-Rojas and Inestrosa, 2018). APP physically interacts with β-catenin; an increase in β-catenin occurs with an increase in APP that potentiates Wnt/β-catenin signaling (Elliott et al., 2018; Zhang et al., 2018). Therefore, APP could contribute to increased brain matter via Wnt/β-catenin signaling.

FMRP inhibits mRNA translation, and metabotropic glutamate receptor (mGluR) signaling regulates FMRP activity. For example, FMRP inhibits a PI3K enhancer (PIKE) which represses mTOR under normal conditions (Huber et al., 2015). FMRP is lacking in FXS (and in some cases of ASD), favoring PIKE stimulation of mTOR and resultant stimulation of Rho family members Rac1 and cofilin that trigger actin disassembly leading to myelination (Nawaz et al., 2015). Is this the mechanism underlying enlarged head circumference and increased brain white matter seen in FXS (Hoeft et al., 2010) and some forms of ASD? Westmark and Malter (2007) found FMRP directly regulates APP through mGluR5 in a mouse model of FXS. APP mRNA is increased in FXS when the FMRP “brake” is released. Consistent with these animal findings, we found increased levels of APP total, sAPPα, and Aβ in plasma from children with FXS, compared to children with ASD and typical development. We found this same APP profile in frontal and temporal lobe samples from adults with FXS (Ray et al., 2016). Of note, the elevation in Aβ seen in FXS plasma and brain tissue was not seen in ASD plasma and brain tissue. In fact, Aβ levels were lower in ASD plasma and brain tissue (Sokol et al., 2006; Ray et al., 2016). Therefore, APP metabolites, in a yet to be defined mechanism, but in a disorder-specific manner, may induce brain white matter expansion in FXS.

## Can hypermyelination cause increased/disrupted white matter in ASD?

OPC processes expand their thin membrane filaments to thick, lipid-filled layered tubes wrapped around axons during differentiation and myelination (Hughes and Appel, 2016). Myelin sheaths growth occurs via two steps. First, an actin network upholds myelin’s leading edge, securing the edge between the axon and its covering oligodendrocyte. Second, actin disassembly helps the myelin membrane unfurl and creep along the axon (Nawaz et al., 2015). Actin is disassembled by cofilin and gelsolin proteins, which favors myelination. Myelin Basic Protein (MBP) is essential in myelination because it releases cofilin and gelsolin from the membrane, thereby deactivating actin. Rho GTPases, RhoA, and Rac1 are distributed via OCP NG2 and lie on either end of the OCP. Rac1 promotes cofilin phosphorylation which in the normal state inactivates cofilin, supporting actin (opposing myelination; Hughes and Appel, 2016). Rac1 has been found to be defective (promoting myelination) in a mouse model of FXS (Chen et al., 2010). Finally, Wnt signaling guides OPC embryonic development toward migration as OPCs traverse endothelial cells (Tsai et al., 2016).

Myelination requires a significant amount of energy. Unsurprisingly, the mTOR pathway, a key regulator of cell metabolism, enables myelination through the mTORC1 hub (Figlia et al., 2018). mTORC1 synthesizes lipid, a major component of myelin. Further, mTORC1 differentiates oligodendrocytes from OCPs and coordinates lipid and protein synthesis to generate the membrane. Interestingly, hypomyelination in the PNS and CNS occurs in TSC due to the deletion of TSC1 or TSC2, which disrupts the TSC complex (Lebrun-Julien et al., 2014; Carson et al., 2015; Jiang et al., 2018). TSC is considered one of the single gene syndromes associated with ASD. Hypomyelination, however, is inconsistent with the TSC phenotype of macro/megalencephaly, increased white matter, and hamartomas seen in individuals with TSC. Therefore, other factors, i.e., APPα/α-secretase activation, besides overactivation of the mTOR pathway favor excessive white matter, as seen in TSC, and ASD.

Alternately, APP dysregulation could be a side effect of neuronal dysfunction associated with genetic conditions such as TSC. Activation of mTOR causes activation of β-secretase, resulting in increased Aβ peptide (Cai et al., 2015). Further, studies of apoptotic and behavioral sequelae of mild brain trauma may explain the biochemical pathway involving APP metabolites (Tweedie et al., 2007). Could Aβ peptide, associated with apoptosis and brain atrophy in AD, contribute to hypomyelination in TSC?

## Proposed translational pathway linking APP to hypermyelination in ASD

**Figure 1:**
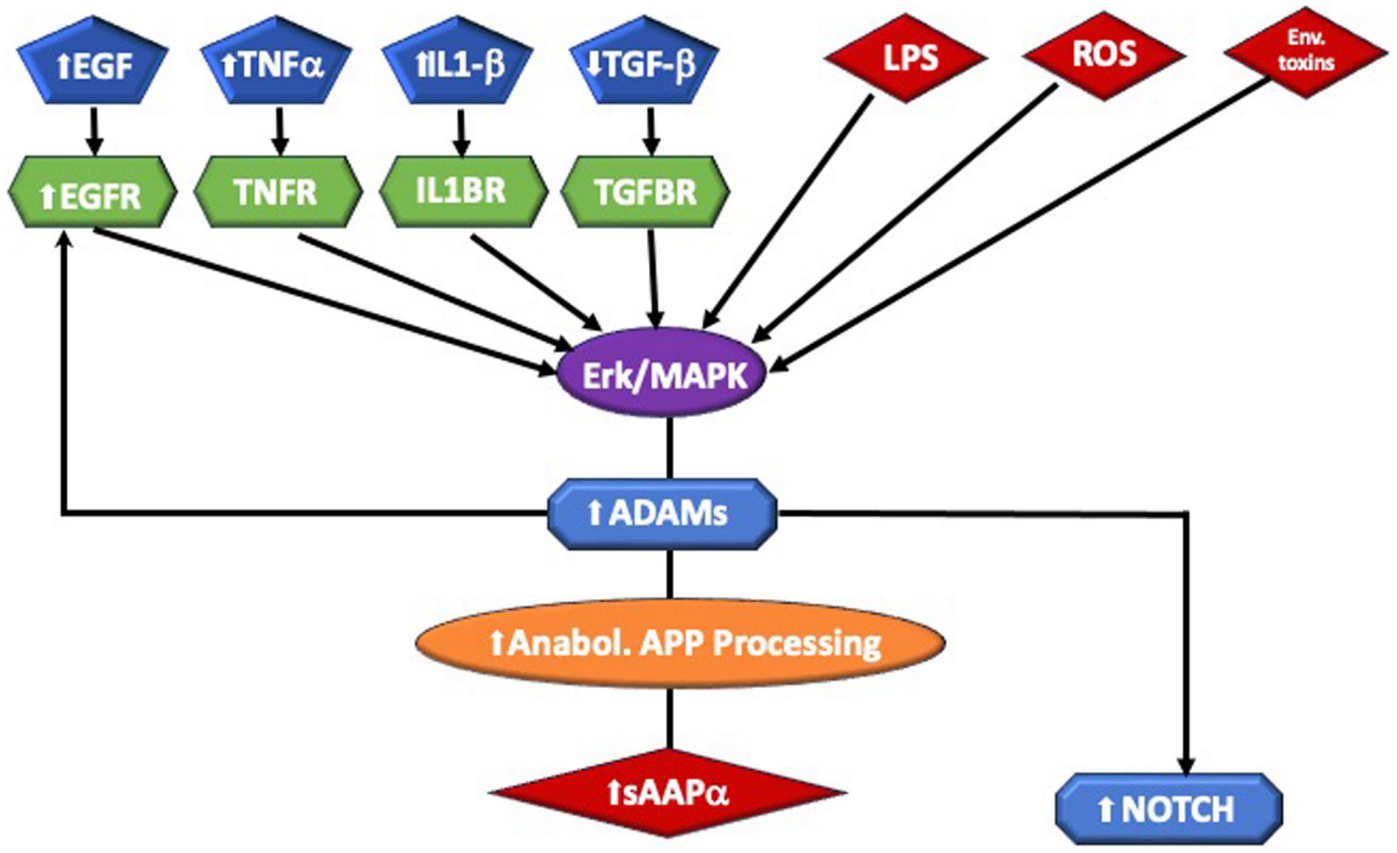
Highlights of sAPPa pathway and putative biochemical mechanism leading to Macrocephaly. Inflammation, infection, and toxins are among the triggers of α-secretase, which are members of the ADAM (A Disintegrin And Metalloprotease domain) family expressed on the surfaces of cells. Cleavage of APP by α-secretase results in the generation of large extracellular sAPPα. ADAM 10 is essential for Notch signaling. Aberrant signaling of the ERK/MAPK pathway may lead to white matter brain overgrowth in ASD. This includes Interleukin 1-beta (IL1β), Endothelial growth factor (EGF) and receptor (EGFR), and Tumor Necrosis Factor-alpha (TNFα,). which are elevated in ASD. External stressors, such as lipopolysaccharide (LPS) and reactive oxidizing species (ROS), generated by oxidative stress, have been connected to ASD. Perturbation of the ERK/MAPK pathway, by a number of causes, can stimulate the ADAM enzymes. ADAMs cleave APP at the α-secretase site, producing sAPPα. They are also required for EGFR signaling and they stimulate NOTCH activity.

We propose an APP pathway contributing to macrocephaly in autism in the following way:

sAPPα→ ERK1/2 → MAPK → PI3K/AKT–mTOR → Rho GTPases → OPC → OL → Hypermyelination (Figure 1).

We provide evidence in support of this pathway.

**Figure 2:**
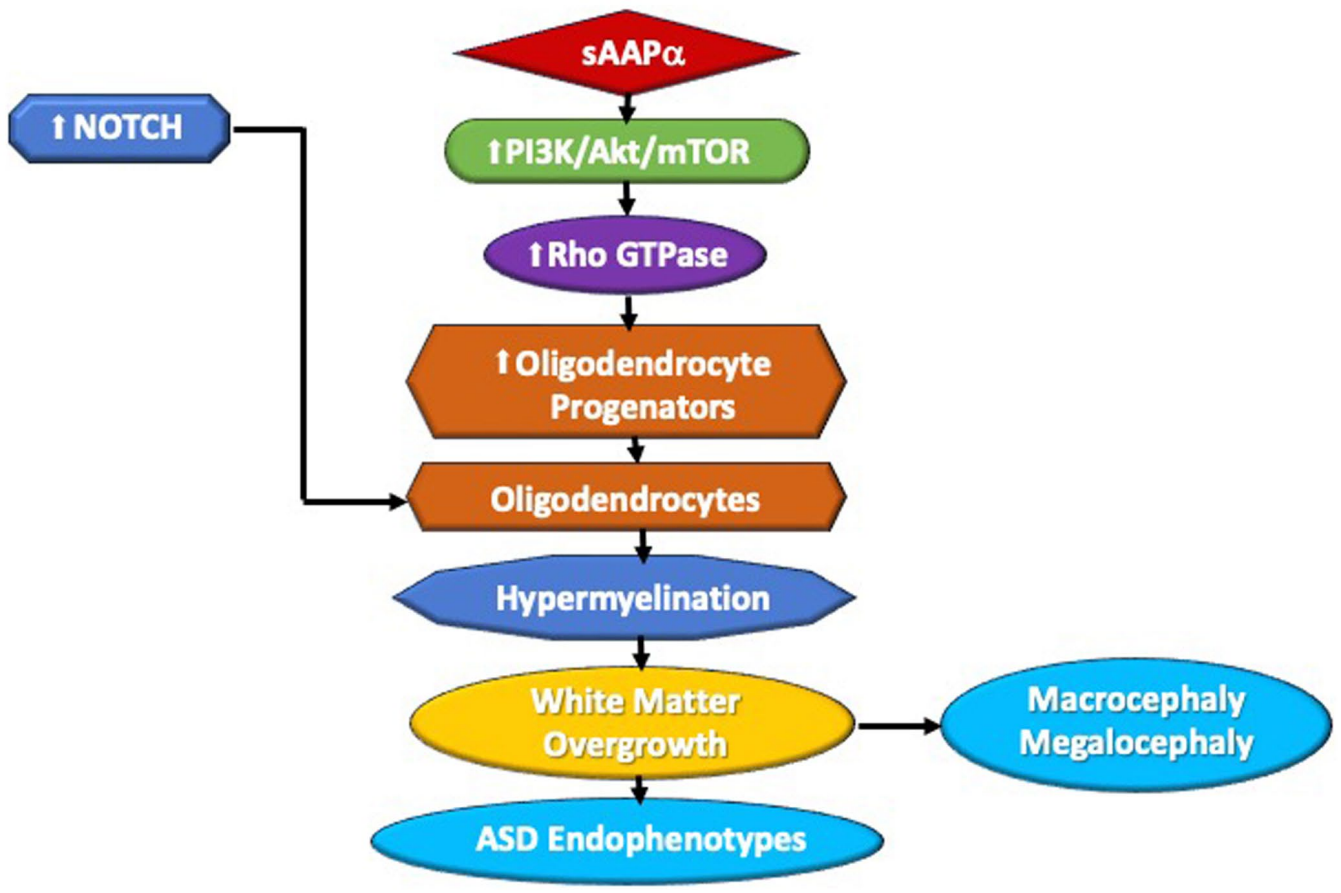
A schematic of sAPPα ‘s potential contribution to macrocephaly in ASD via the PI3K/Akt/mTOR/Rho GTPase translation pathway. Elevated sAPPα enhances PI3K/Akt/mTOR pathway activity, leading to activation of rho GTPase. The GTPase stimulates oligodendrocyte progenitors, which utilize NOTCH activity to mature into oligodendrocytes. Increased oligodendrocytes favor hypermyelination, which would lead to white matter brain overgrowth. The white matter overgrowth would contribute to ASD endophenotypes, i.e., macrocephaly, poor social functioning, or regression.

Normal physiological processing of APP produces far more sAPPα than Aβ peptide, so adamalysins are active in normal development. As cited above, ADAM17 appears directly involved in CNS myelination and oligodendrocyte development (Palazuelos et al., 2015). In addition, sAPPα appears to directly affect myelin: an *in vitro* study in mouse cerebellum showed that sAPPα activator etazolate protected myelinated axons from demyelination and increased the number of mature oligodendrocytes (Llufriu-Dabén et al., 2018). BDNF boosts OPC proliferation and growth via the TrkB and MAPK pathways (Van't Veer et al., 2009), and promotes the α-secretase release of sAPPα (Gao et al., 2022). However, other triggers that activate ERK could increase sAPPα via ADAM17. For example, a prenatal insult, toxins, or inflammation could increase the expression of NMDA and glutamate receptors. Increased numbers of glutamate receptors activate the ERK signaling cascade. ERK1/2 controls the thickness of CNS myelin once oligodendrocyte differentiation and myelin initiation take place (Ishii et al., 2012); sAPPα stimulates phosphorylation of ERK 1/2 (Cheng et al., 2002) ERK, and p38Map kinase activate ADAM17, favoring sAPPα (Xu and Derynck, 2010; Arkun and Yasemi, 2018). sAPPα has been shown to activate PI3K/AKT–mTOR (Cheng et al., 2002), resulting in aberrant brain growth. These signaling pathways may impact both gray and white matter. As described above, however, mTOR pathways tend to favor excess myelination. Akt activation of the PI-3-kinase pathway stimulates mTORC2, which controls and maintains the actin cytoskeleton via Rho GTPace family members RhoA and Rac1 signaling (Biname et al., 2013). Rac1 activates cofilin that favors the disassembly of actin leading to myelination, again stimulated by α-secretase. Further, APP metabolites may influence myelination in other ways. Netrin-1 appears to underlie OPC density and turnover (Birey and Aguirre, 2015), and WNT signaling supports OPC expansion along blood vessels (Tsai et al., 2016). APP regulates Netrin-1 mediated commissural axon outgrowth (Rama et al., 2012) and Wnt signaling protects against the effects of Aβ peptide, favoring the nonamyloidogenic pathway (Tapia-Rojas et al., 2016). Therefore, other potential pathways enable APP regulation of myelination (Figures 1, 2).

## Summary: APP in ASD macrocephaly

Brain enlargement in ASD may be caused by neuroinflammation or hyperactivated signaling translational pathways. Increased sAPPα and the adamalysins may directly increase oligodendrocyte myelination or the neuroinflammatory response that promotes axonal sprouting of neurons and astrocyte activation. APPα, and the adamalysins, activated by ERK receptor signaling, may in turn activate PI3K/Akt/mTOR and then Rho GTPases that stimulate OPC, increasing myelination by activating cofilin. Disrupted miRNAs may increase adamalysins, increasing sAPPα, and favoring excessive brain growth in ASD. The potential contribution linking the nonamyloidogenic pathway to brain enlargement in ASD enables the novel adaptation of pathways long known in AD to ASD. This theory suggests a possible molecular connection between two ends of life diseases: ASD and AD.

## Author contributions

DS: original draft preparation. DL: critical revision of the manuscript. All authors contributed to the article and approved the submitted version.

## Conflict of interest

The authors declare that the research was conducted in the absence of any commercial or financial relationships that could be construed as a potential conflict of interest.

## Publisher’s note

All claims expressed in this article are solely those of the authors and do not necessarily represent those of their affiliated organizations, or those of the publisher, the editors and the reviewers. Any product that may be evaluated in this article, or claim that may be made by its manufacturer, is not guaranteed or endorsed by the publisher.
